# Materials Enabling Methane and Toluene Gas Treatment

**DOI:** 10.3390/ma17020301

**Published:** 2024-01-07

**Authors:** Tong Lv, Rui Wang

**Affiliations:** School of Environmental Science and Engineering, Shandong University, Qingdao 266237, China

**Keywords:** adsorption, catalytic oxidation, hydrogen reforming, non-oxidative coupling, toluene, methane

## Abstract

This paper summarizes the latest research results on materials for the treatment of methane, an important greenhouse gas, and toluene, a volatile organic compound gas, as well as the utilization of these resources over the past two years. These materials include adsorption materials, catalytic oxidation materials, hydrogen-reforming catalytic materials and non-oxidative coupling catalytic materials for methane, and adsorption materials, catalytic oxidation materials, chemical cycle reforming catalytic materials, and degradation catalytic materials for toluene. This paper provides a comprehensive review of these research results from a general point of view and provides an outlook on the treatment of these two gases and materials for resource utilization.

## 1. Introduction

Greenhouse gases (GHGs) and volatile organic compounds (VOCs) are by far the two most damaging groups of substances to ecosystems, and methane and toluene are the typical representatives of these two groups, respectively. The treatment of methane and toluene represents, to some extent, the treatment of GHGs and VOCs. Methane is the hydrocarbon with the lowest carbon content and is the world’s second-largest greenhouse gas after carbon dioxide. Although the atmospheric content of methane is much smaller than that of carbon dioxide, methane absorbs atmospheric thermal infrared radiation much more efficiently than carbon dioxide, and its global warming potential (GWP) per unit mass is 86 times higher than that of carbon dioxide over 20 years [1]. Therefore, the emission of methane into the air causes a serious greenhouse effect, which in turn threatens the human environment. Toluene is a colorless liquid with a specific aromatic odor and strong volatility. It is classified as a Class 3 automotive carcinogen according to the World Health Organization (WHO) list of carcinogens. Methane poses a serious threat to the environment as it pollutes the air, atmosphere, and water. Excessive emission of methane and toluene into the environment will therefore adversely affect human health and the ecological environment, and their emission must be restricted and properly handled. At the same time, these two gases have a certain value of resource utilization, which can be achieved if these two substances are treated by reasonable means. In the case of methane, in addition to adsorption and storage for reuse, we can use catalytic oxidation to convert it into methanol and other important raw materials for production and utilization, as well as decomposition and reforming to produce hydrogen. In addition, the aromatic coupling of methane in the absence of oxygen to produce aromatic compounds for industrial production is also an effective way to treat and create resources from methane. As for toluene, catalytic oxidation, catalytic decomposition, and chemical cycle reforming are common treatment methods. For the removal and resource utilization of these two substances, researchers have carried out many studies, in which the treatment of these two substances’ auxiliary materials is crucial. This research area is currently one of intense interest. This paper categorically outlines and summarizes the latest research results on treatment aids for methane and toluene over the past two years and looks at future directions for these materials.

## 2. Materials for Methane Handling

### 2.1. Methane Adsorption Materials

**Figure 1:**
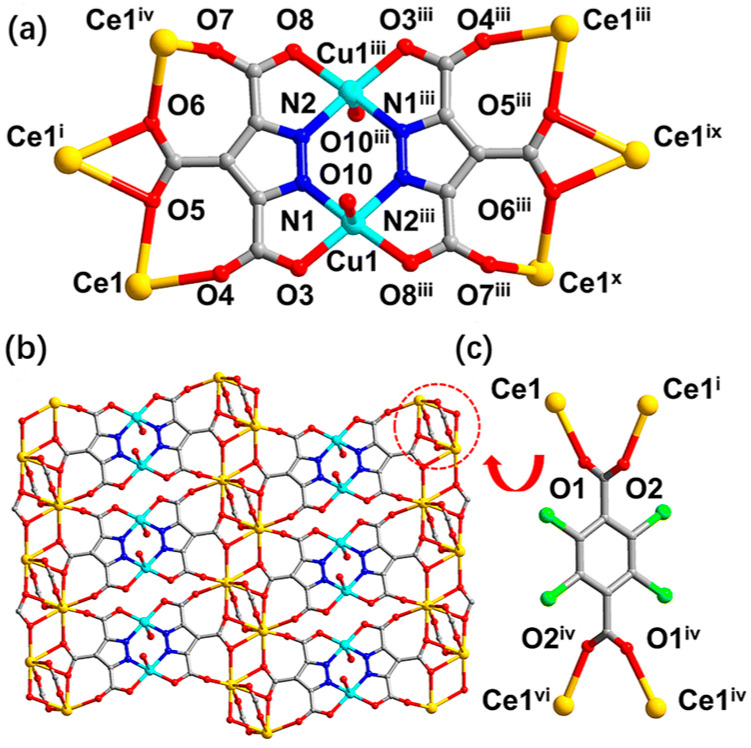
(**a**) Coordination pattern and connectivity of the Cu ion and L ligand. (**b**) 3d-4f Cu-Ce layer bridged by L ligand. (**c**) Coordination mode of the bridged Cl_4_-bdc ligand. Symmetry codes: ^i^ 2 − *X*, 1 − *Y*, 1 − *Z*; ^ii^ 1 + *X*, +*Y*, +*Z*; ^iii^ 1 − *X*, 1 − *Y*, −*Z*; ^iv^ −1 + *X*, +*Y*, +*Z*; ^v^ 2 − *X*, 2 − *Y*, 1 − *Z*; ^vi^ +*X*, 1 + *Y*, +*Z*; ^vii^ 3 − *X*, 1 − *Y*, 1 − *Z*; ^viii^ 2 + *X*, +*Y*, +*Z*; ^ix^ −1 + *X*, +*Y*, −1 + *Z*; ^x^ 2 − *X*, 1 − *Y*, −*Z* [3].

**Figure 2:**
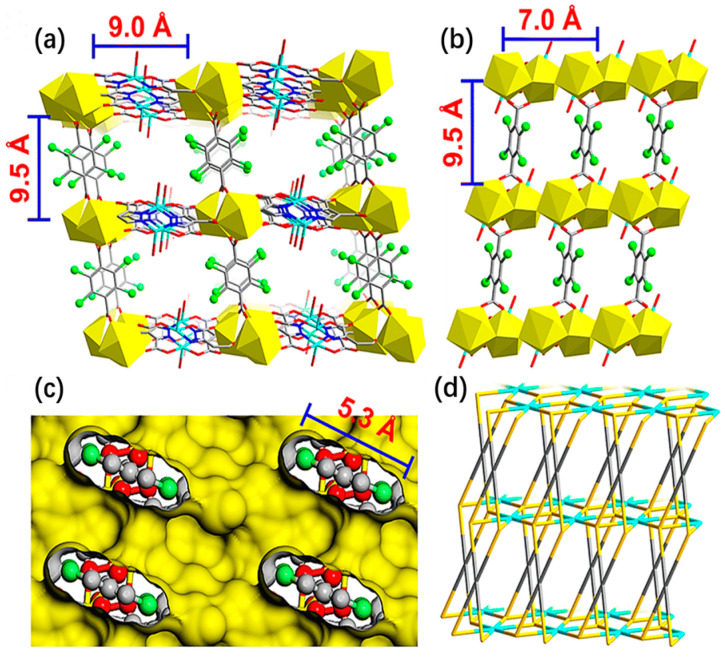
(**a**,**b**) Three-dimensional structural view of MOF[CuCeL(Cl_4_-bdc)_0.5_(H_2_O)_2_(H_2_O)_6_]_n_ along the a- and c-axes (all free solvent molecules have been removed to clearly show the pore structure). (**c**) Internal structure of the pore along the b-axis with a yellow Connolly surface. (**d**) 1 Morphology along the c-axis. The atoms and structures represented by each color in the figure are the same as in Figure 1 [3].

Adsorption has been widely used for methane collection and treatment as a simple and economical methane gas treatment method, and methane adsorption materials constitute a popular research topic. Among them, metal-organic frameworks (MOFs) can be used as a good methane adsorption material, which can adsorb and store methane efficiently at moderate temperatures and pressures. Wu and Zhou found that MOFs with moderate interactions with methane and high densities have the greatest working capacity in natural gas storage, and that the flexible characteristics of some frameworks give these MOFs a high working capacity [2]. A multicomponent MOF[CuCeL(Cl_4_-bdc)_0.5_(H_2_O)_2_ (H_2_O)_6_]_n_ (L = 1H-pyrazole-3,4,5-tricarboxylic acid, Cl_4_-bdc = 2,3,5,6-tetrachloroterephthalate) with a column-layer structure was studied by Zhu et al. [3]. Figure 1 and Figure 2 show schematic diagrams of the coordination patterns and connections of the catalytic material and the three-dimensional structure of the stereo.

The material has a high adsorption capacity of 28.41 cm^3^/cm^3^ for CH_4_ and only 3.43 cm^3^/cm^3^ for N_2_ (298 K, 1 bar), which provides a good methane–nitrogen separation.

Activated carbon is an important methane adsorption material, and its pore structure has an important influence on the methane adsorption effect. When biomass or biochar is activated by KOH, the necessary hierarchical porous network structure is formed, and the pore structure produced by different biomasses is different. Adlak et al. found that activated carbons made from coconut shells and pistachio shells had the proper size pore structure required for methane adsorption, but activated carbons prepared from other softer biomasses tended to have larger pore structures [4].

In order to examine the impact of activators on the pore opening of activated carbon (AC), Zaini et al. synthesized AC from palm kernel shell (PKS) using three different activators: steam, carbon dioxide, and carbon dioxide–steam. It was discovered that the PKS-made AC had a significant amount of methane adsorption potential. With a maximum adsorption capacity (MAC) of 4.5 mol/kg for methane gas (MG) and a specific surface area (SSA) of 869.82 m^2^/g, the ACs produced from CO_2_-steam exhibited varying rates of molecular diffusion and reabsorption. Additionally, their total pore volume (TPV) was measured at 0.47 cm^3^/g. The Freundlich isotherm model fits all kinds of ACs extremely well, suggesting the emergence of multilayer adsorption (MLA). The formation of multilayer adsorption is indicated by this, and the adsorption kinetic data of the generated ACs conform to the pseudo-first-order model, in which the adsorption rate is primarily dictated by the physical adsorption between the pore surfaces and the methane gas, and the simultaneous influence of pore diffusion and outer diffusion on the adsorption of methane [5]. By thermochemically activating wood waste in H_3_PO_4_ at 1173 K, Pribylov et al. created the ES-103 microporous carbon adsorbent. At 303 K and 20 MPa, the adsorbent’s methane adsorption capability was 22 weight percent. At 303 K and 20 MPa, the adsorbent’s methane adsorption capability was 22 weight percent. Following the compaction of the ES-103 adsorbent with a binder, the bulk density of methane at a pressure of 10 MPa was 200 m^3^NTP/m^3^ [6].

**Figure 3:**
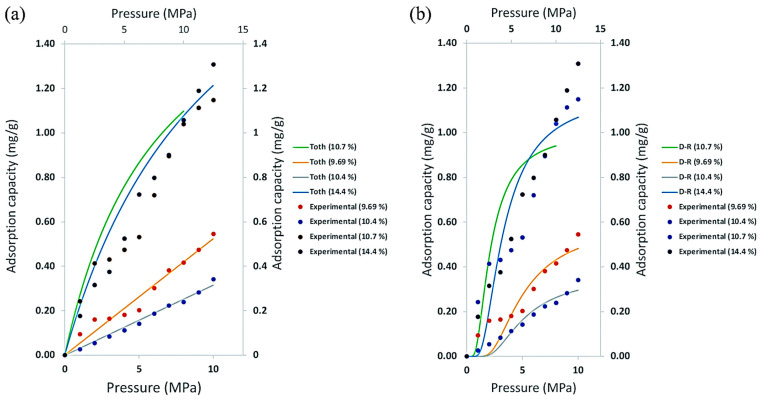
(**a**) Toth isotherm; (**b**) D-R isotherm [8].

Shale is a rock formed by dehydration and cementation of clay. It is dominated by clay minerals and has a unique thin-layered structure. Organic matter, also known as kerogen, is present in shale and is a microscopic structure with physical properties that are very different from the other components of shale. Despite their micron- and nanometer-scale composition, shales can adsorb and store large amounts of gases, thanks to the very large surface area of the cuticle. The use of shale for the adsorption and storage of methane and carbon dioxide is a promising technology for both reducing greenhouse gas emissions and improving the resource utilization of methane [7]. Adsorption isotherms of shale materials are important in understanding the mechanism of gas storage in shale. With the aid of isothermal adsorption experiments, Aji et al. examined the effects of total carbon (TOC), pore size distribution, and mineralogical properties on the adsorption capacity of shale. They also measured methane adsorption isotherms using gravimetric adsorption at a temperature of 120 °C and a maximum pressure of 10 MPa on four shale core samples from the Eagle Ford reservoir. The article’s plots, like Figure 3, illustrate the relationship between the samples’ measured TOC, adsorption capacity, and adsorption isotherms. Supercritical methane’s calculated absolute adsorption capacity is higher than its excess adsorption capacity. At pressures greater than 9.6% of the critical methane pressure, the discrepancy between the absolute and excess adsorption capacities is more pronounced [8].

In order to investigate the methane absorption, desorption, and diffusion capacities in various coal samples, Zuo et al. employed both experimental and molecular simulation techniques. They then suggested an enhanced fracturing fluid formulation consisting of 0.8% CATB + 0.2% NaSal + 1% KCl + SiO_2_ [9].

Compared with the coal sample treated with clean fracturing fluid, the methane adsorption capacity and desorption capacity of the coal sample treated with nano-fracturing fluid are improved to a certain extent. In addition, nano-particle-modified clean fracturing fluid can also reduce the damage caused by clean fracturing fluid to the desorption and diffusion capacity of coal seam. In addition, some studies on the effectiveness of some other materials for methane adsorption have been performed. David Ursueguia and colleagues produced composites consisting of HKUST-1 and Al_2_O_3_ particles, and evaluated their ability to adsorb methane. The samples were placed on a fixed bed and aged in the air at 100% relative humidity for 24 h in three successive cycles in order to test the materials’ ability to adsorb methane both before and after wet treatment. It was demonstrated that the adsorption capacity of the composite with lower MOF loadings (<9%) rose by more than 38%, while the methane adsorption capacity of HKUST-1 decreased by around 37% [10].

### 2.2. Catalytic Oxidizing Materials

**Figure 4:**
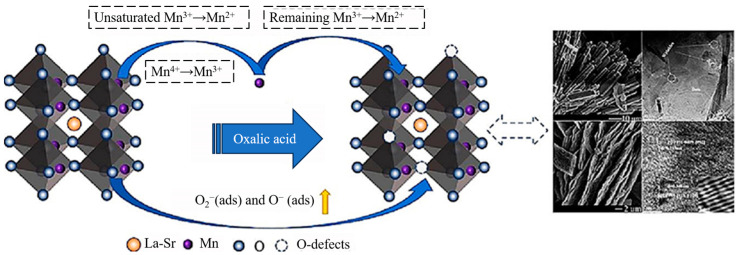
Catalyst structure and reaction diagram [11].

Methane poses environmental hazards and depletes resources; however, these problems can be effectively mitigated through the development of cost-effective catalysts and reactors for methane treatment and resource utilization. Zhao et al. created a unique non-precious metal catalyst for the catalytic oxidation of methane using oxalic acid etching of La_0.8_Sr_0.2_MnO_3_ [11]. Figure 4 depicts a schematic design of the process of this catalyst’s catalytic oxidation of methane.

The catalyst etched with oxalic acid catalyzed the methane oxidation more efficiently than the unetched sample. Khatun et al. prepared a defect-rich Ni-Pt/CeO_2_ catalyst by adding nickel and platinum to the ceramic lattice using a one-pot composite combustion process. The catalyst has excellent catalytic activity and stability, and can catalyze the reaction stably for up to 700 h. In this process, the conversion of CH_4_ and CO_2_ was close to 86% [12].

**Figure 5:**
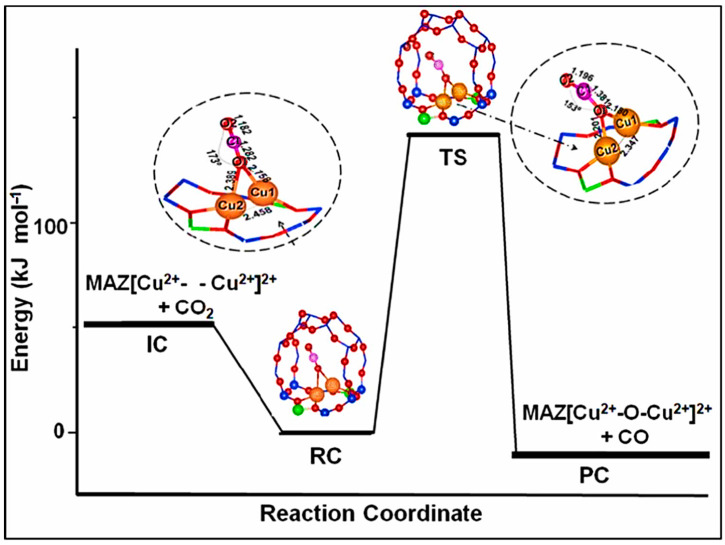
Calculated energy diagrams of carbon dioxide activation at sites. IC: initial complex, RC: reactant complex, TS: transition state, PC: product complex, where only the active site and zeolite pores are shown and other atoms of the zeolite are omitted. Blue (Si), red (O), green (Al), orange (Cu), and magenta (C) [17].

Through catalytic oxidation, methane is converted to methanol, which not only lessens methane’s impact on the atmosphere but also yields methanol, a crucial raw material for both residential and industrial manufacturing. In their study on methane methanolization, Alvarez et al. discovered that the productivity of methanol is influenced by the adsorption period, and that it takes at least 60 min for the adsorption to reach equilibrium. Methane chemisorption’s equilibrium and kinetics were modeled based on the synthesis of two adsorption precursors: carbon dioxide and methane. Additionally, they looked at the aerobic methane adsorption process to apply methane conversion to the methanol cycle in these situations [13]. Vitillo et al. compared the reaction profiles of four single iron-based catalysts for the direct oxidation of methane to methanol using two biomimetic models based on two enzymes (cytochrome P450 and taurine dioxygenase [Taub]) and two artificial reticulation frameworks (iron–BEA zeolites and a triple-ferric oxide-centered metal-organic frameworks) using the Kohn–Sham density functional method. When methane conversion was more than 1%, the biomimetic and inorganic catalysts’ selectivity for methanol was nearly nonexistent at room temperature. The authors stressed that in the event of a lack of a methanol protection method, attaining high selectivity necessitates simulating the enzyme’s reaction milieu beyond the initial iron coordination layer [14]. A metal-organic framework Fe/UiO-66 supports an iron catalyst, which allows methane to be selectively oxidized to methanol, as reported by Rungtaweevoranit et al. At 180 °C, methanol was consistently generated with strong selectivity for methanol at an excellent reaction rate of 5.9 × 10^−^^2^ μmol_MeOH_g_Fe_^−1^s^−1^ [15]. Zhu et al. doped bismuth oxychloride (BiOCl) with non-precious nickel sites and gave it a high oxygen vacancy content, which allowed them to directly oxidize CH_4_ to CH_3_OH in a single step. As methane oxidized to create methyl and adsorbed hydroxyl groups, the oxygen vacancies of the unsaturated Bi atoms adsorbed and activated CH_4_, keeping the catalyst active. As much as 39.07 μmol/(gcat-h) of O_2_ and H_2_O-based CH_3_OH was converted at 420 °C and under flow conditions [16]. Pereira et al. developed a new process using copper-exchanged zeolite omega (Cu-MAZ) by discovering that carbon dioxide can be used as a substitute for oxygen and that CH_3_OH yields are higher when carbon dioxide is involved in the reaction. The calculated energy diagram for carbon dioxide activation at the site is shown in Figure 5 [17].

To further maximize the selectivity of methane oxidation to methanol, Zhou et al. generated 12 single-atom alloys (SAAs) for methane activation and screened those with good C-H bond dissociation catalytic activity. The methane dissociation activity is enhanced when Ir metal atoms are doped in inert covalent matrices (Ag, Au, and Cu). Ir1/Ag SAA is the most effective single-atom amalgam catalytic element for selectively oxidizing methane to methanol. This work offers crucial guidance for the future development of extremely active and effective methane methanolization catalysts [18]. Using several bismuth-based catalysts (BiPO_4_, α;-Bi_2_O_3_, β;-Bi_2_O_3_) and molecular oxygen as the only oxidant, Matsuda et al. studied the direct oxidation of methane (CH_4_) to formaldehyde (HCHO). When it came to the direct oxidation of CH_4_, the monoclinic BiPO_4_ nanoparticles (BiPO_4_-DEG) that were created in diethylene glycol (DEG) and water-mixed solvent exhibited the maximum catalytic activity. BiPO_4_-DEG outperforms FePO4 nanoparticles in the high-temperature range, where the reactive oxygen species on the surface of BiPO_4_ combines with CH_4_ to form HCHO, while FePO4 nanoparticles form HCHO for the phosphate units therein that react with CH_4_ [19]. A proton-type zeolite catalyst containing Bronsted acid sites that are appropriate for CH_4_ combustion was predicted and shown to work very well by Yasumura and associates. They investigated main-group elemental catalysts using silicon and aluminum for low-temperature CH_4_ combustion with ozone, based on automated reaction route mapping. At 250 °C, it was discovered that catalysts containing strong Brønsted acid sites improved methane conversion. These catalysts show promise for methane combustion at low temperatures. At 190 °C, the benchmark catalyst (5 weight percent Pd-loaded Al_2_O_3_) catalyzed a reaction rate 442-fold lower than the main group catalyst (protonated β-zeolite). In light of autonomous reaction route mapping, this illustrates the sensible design of an earth-rich catalyst [20]. Using iron or copper oxides as essential intermediates, related researchers have created a variety of biomimetic molecular catalysts that are modeled after methane monooxygenases (MMOs). They are still far less effective than MMOs for catalyzing the oxidation of methane, though. In order to generate methane oxidation catalytic materials with better catalytic activity, Yamada et al. firmly stacked μN-bridged iron phthalocyanine dimers onto graphite surfaces. This graphite-supported μ-nitrogen-bridged iron phthalocyanine dimer could oxidize methane even at room temperature, and its catalytic activity was comparable to that of MMO, nearly 50 times higher than that of other powerful molecular methane oxidation catalysts in an aqueous solution containing H_2_O_2_ [21]. They also created a new mu-nitrogen-bridged heterodimer of iron porphyrin and iron phthalocyanine, and studied its catalytic CH_4_ oxidation characteristics. When H_2_O_2_ was present in acidic aqueous solutions at 60 °C, the heterodimers demonstrated catalytic activity for CH_4_ oxidation via high-valency iron–oxygen species. The μ-nitrogen-bridged iron porphyrin dimer’s high-valency iron–oxygen species is extremely unstable under the same reaction circumstances [22]. Several CoxCeMgAlO mixed oxides with varied cobalt contents of 10% Ce and Mg/Al atomic ratios of 3 were obtained by Stoian et al. calcining layered double hydroxide (LDH) precursors at 750 °C. The complete oxidation of methane was used to assess their catalytic qualities. Because of their optimal redox characteristics and maximum Co/Ce surface atom ratio, the Co_40_CeMgAlO mixed oxides exhibited the highest catalytic activity for methane combustion out of all of them. They also demonstrated the good stability of the catalytic activity by examining the impact of contact time [23]. Larger reaction temperatures are usually needed for the oxidative coupling of methane (OCM) in order to achieve larger C_2_ yields; nevertheless, methane may be severely oxidized, leading to lower C_2_ yields. Deep oxidation of methane causes CH_3_ surface dimerization, which must be eliminated with a lower catalytic reaction temperature. In order to better understand OCM catalytic performance, Maulidanti et al. constructed M/Ce(Y) catalysts. The greatest catalytic performance was reported at 5% Fe_3_O_4_/CeO_2_(A), with a 2:1 CH_4_/O_2_ ratio, 0.5 g of catalyst, and 27% CH_4_ conversion and 1.4% C_2_ production at 500 °C. Reducing the CH_4_/O_2_ ratio and raising the catalyst weight will increase the C_2_ yield. Fe_3_O_4_ and CeO_2_ together provide a large specific surface area and mesoporous pore size, which is highly useful for OCM at low temperatures [24].

### 2.3. Catalytic Materials for Dry Methane Conversion

In the hydrogen from the methane-reforming (DRM) process, methane and carbon dioxide can be simultaneously converted into syngas with H_2_ and CO ratios close to 1, which provides a promising green pathway for large-scale utilization of methane in the chemical production of hydrogen. However, the industrialization of DRM has been seriously hindered by the sintering of nickel and carbon deposition in the process. Several scientists have made great efforts to solve this problem. By preparing a variety of uniformly distributed Fe-coated Ni/Al_2_O_3_ catalysts using atomic layer deposition (ALD), Zhao et al. found that adjusting the molar concentration of trace amounts of Fe (0.3–0.6%) could improve the low-temperature catalytic activity, accelerate the oxidation process of coke, and promote the dissolution of CH_4_ on NiO. The activity of the 0.3% Fe/Ni/Al_2_O sample was essentially unchanged for 72 h at 650 °C. The activity of the 0.3% Fe/Ni/Al_2_O sample was found to be very low [25].

The modification of Fe achieved the dual effect of enhancing Ni/Al_2_O_3_-catalyzed CH_4_ cracking and eliminating coke. Yadav et al. prepared monometallic (nickel, cobalt) substitutes and CeO_2_ catalyst carriers using solution combustion synthesis and formaldehyde reduction methods. The DRM activity of the catalysts was significantly different. The co-substituted CeO_2_ catalysts showed maximum stability in the DRM reaction at 800 °C. The catalysts showed significant stability. The catalysts showed significant stability. In addition, they found that the presence of graphitic and amorphous carbon was responsible for the deactivation, while the reactivity of surface lattice oxygen played an important role in the catalytic cracking stability, which in turn determined the steps involved in the process. The energy required to generate vacancies in cobalt-substituted CeO_2_ is much lower compared to nickel-substituted CeO_2_. It can therefore be confirmed that while nickel inhibits oxidation by decreasing the availability of surface oxygen required for the reaction, Co-substituted catalysts promote oxidation by increasing the availability of surface oxygen [26]. Density functional theory was utilized by Bandurist and Pichugina to simulate the methane dry-reforming process, which involves the breakdown of C-H bonds on Cu-rich Ni-Cu clusters. The nanoscale clusters NiCu_11_S_6_(PH_3_)_8_, NiCu_11_S_6_, NiCu_11_O_6_(PH_3_)_8_, and NiCu_11_O_6_ were used to simulate the catalysts. Based on the collected data, the most promising catalytic system for CH_4_ activation was determined to be NiCu_11_O_6_, with an activation energy of 99 kJ/mol. This system also showed the best thermodynamic performance [27].

**Figure 6:**
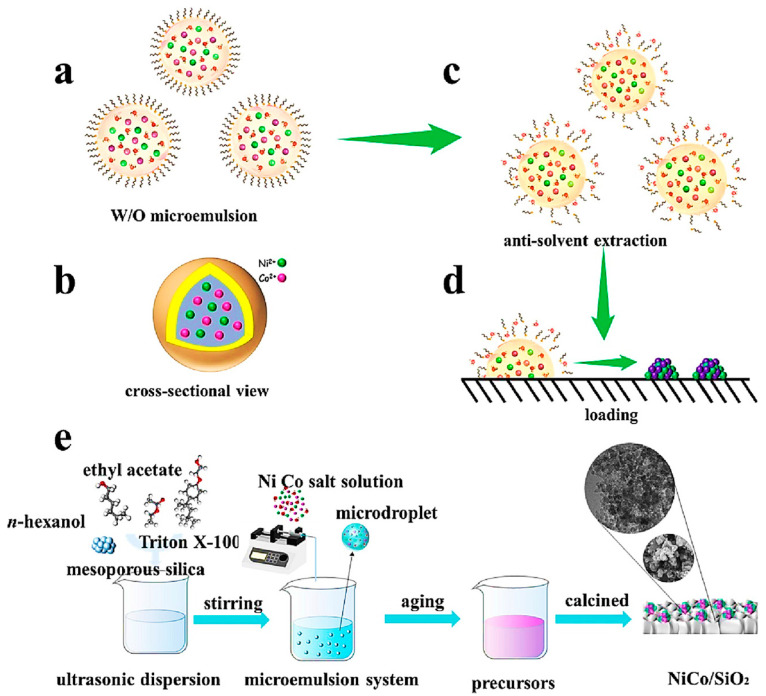
Schematic diagram of the catalyst synthesized by the microdroplet method [28]. (**a**) Water-in-Oil Microemulsion Droplet; (**b**) Cross-sectional view of water-in-oil microemulsion droplets; (**c**) Gradual diffusion of water from the droplets into the ethyl acetate phase; (**d**) Co-precipitation of precursor salts by microemulsion systems disrupted by water loss; (**e**) Catalyst preparation flow chart.

Zou et al. prepared a novel nickel–cobalt nanocomposite catalyst, and the synthesis process is schematically shown in Figure 6. Compared with the monometallic catalysts (10Ni0Co/SiO_2_, 0Ni10Co/SiO_2_), the bimetallic 5Ni5Co/SiO_2_ nanocomposite catalysts showed the best catalytic activity, where the 10Ni0Co/SiO_2_ catalysts showed a 10% decrease in catalytic activity due to coking in the 50 h catalytic experiment. The 5Ni5Co/SiO_2_ bimetallic catalyst maintained its catalytic activity within 100 h. The catalytic activity of the 5Ni5Co/SiO_2_ bimetallic catalysts was also improved [28].

A series of Ni/MSS catalysts were prepared using four different methods (normal impregnation, glycine-assisted impregnation, glycol-assisted impregnation, and ammonia evaporation) such as that of Zhang. Among these methods, except for the catalysts prepared by normal impregnation, the other three methods, especially glycine-assisted impregnation, and ethylene glycol-assisted impregnation, could obtain smaller nickel particles, improve carrier–metal interactions, and effectively increase the coking and sintering resistance of the catalysts. In addition, the catalyst prepared by the ammonia evaporation method has the best stability [29]. On the other hand, Zhang et al. constructed NiMgAlOx/BN catalysts with closed interfaces (NiMAO/BN) using layered metal oxides (NiMgAlOx) and boron nitride (BN). They found that the triple interface between Ni, BN, and MgAlOx oxides in the catalyst enhanced the sintering resistance of the catalyst [30]. In addition, Zheng et al. investigated a material combining nickel, the oxygen carrier Ca_2_Fe_2_O_5_, and CaO for a cyclic DMR process. The activated NiO/Ca_2_Fe_2_O_5_/CaO material produced large amounts of syngas and H_2_ during cycling, and the material remained active for more than 30 cycles [31]. In the course of studying how supported precursors affected the catalytic activity of nickel catalysts (20% Ni/5% La_2_O_3_-95% Al_2_O_3_) used in the methane reforming process, Zakrzewski et al. discovered that catalysts made with precursors containing chloride had extremely low methane and carbon dioxide conversion rates [32]. For methane cracking on Ni(111) under steam-reforming circumstances, Yadavalli et al. created an ab initio kinetic Monte Carlo (KMC) model. In a reasonable amount of computing time, the model provides insight into the coking state of graphene/coke by capturing the C-H activation kinetics and characterizing the production mechanism of graphene flakes at the thermodynamic level. They employed ever more fidelity clusters to compare the predictions of a KMC model that integrates these clusters into a mean field micromotion model in order to methodically assess the impact of effective cluster interactions between C and CH species on the coking state. The findings demonstrate that there is a considerable correlation between the coking condition and cluster faithfulness. Furthermore, at low temperatures, C-CH islands/rings are virtually disconnected, whereas at high temperatures, they wrap completely around the Ni(111) surface, according to high-fidelity simulations [33].

In addition to research on catalyst resistance to carbon deposition, there have been advances in the study of new materials for methane reforming catalysis. Hanifa et al. investigated the effects of solvent and pretreatment processes on the Ni catalytic performance of palm oil fuel ash (POFA) catalysts loaded with 10% Ni. The POFA carriers prepared by the ultrasonic pretreatment process had smaller particle sizes compared to the conventional stirring process, and had 71% initial CH_4_ conversion and 4.2% initial H_2_ yield in methanolysis [34]. V_2_CTx/V_2_AlC catalysts modified with nickel were fabricated using impregnation/precipitation and high-frequency etching techniques by Wysocka et al. Their catalytic function was studied at 800 °C and 1 bar in the DRM process. It was shown that the V_2_CTx/V_2_AlC-based material undergoes a phase transition to produce aluminum oxide, vanadium oxide (V_2_O_3_), and vanadium carbide (V_8_C_7_). Nickel is necessary for the catalytic activity of the V_2_CTx/V_2_AlC catalysts. Catalytic activity fluctuations were related to changes in the nickel phase and sodium ion intercalation processes during the preparation process. It was demonstrated that the presence of alkali ions and their distribution of the copper layer affected the catalysts’ stability and activity. The Ni-V_2_CTx/V_2_AlC_IMP catalyst using just the technique of impregnation and no precipitant was found to have the maximum activity and stability in the DRM process. The nickel phase of the catalyst was uniformly distributed and produced a needle-like shape. During a 20 h test, the CO_2_ and CH_4_ conversions were 90–93% and 90–80%, respectively. After 20 h of DRM, the molar ratio of H_2_ to CO in the outflow stream varied from 1.05 during the first hour of the process to 0.95. The unmodified V_2_CTx/V_2_AlC, unetched V_2_AlC, and Ni-SiO_2_ catalysts were less active and less stable than the Ni-V_2_CTx/V_2_AlC_IMP catalysts [35]. Pourghadiri and Sari created an inverted flow microchannel monolithic reactor to improve the enzyme-mediated partial oxygenation of hydrocarbons over Rh/Al_2_O_3_ catalysts for the production of syngas. They used a one-dimensional heterogeneous non-stationary model to simulate the reactor behavior. The full GRI 3.0 mechanism model was used for the gas-phase reactions, and the Langmuir–Hinshelwood surface mechanism was referenced for the catalytic reactions. Investigations were conducted into the impacts of feed preheating temperature, feed CH_4_/O_2_ ratio, reaction pressure, and flow switching time on yield, quality, and methane conversion. The results show that reverse flow operation significantly increases syngas yield and reduces the minimum preheating temperature required for reactor ignition. After the establishment of the cyclic steady state, syngas yields of up to 75% were achieved with feed CH_4_/O_2_ ratios close to 1.6, and H_2_/CO ratios close to 2.7. Compared with unidirectional operation, reverse-flow operation increased methane conversion and syngas yields by at least 8% and 76%, respectively [36].

Apart from the catalyzed dry reforming of methane (DRM) reaction, another process that is more likely to achieve thermodynamic equilibrium and yield higher feedstock gas conversions without carbon displacement is heating plasma-activated replication (CRM) of CH_4_-CO_2_ to the syngas under non-catalytic conditions. Zhou et al. investigated the conversion process and optimum situation of a hot plasma-activated CRM reaction system. As the CO_2_/CH_4_ molar ratio rose, the CO_2_ conversion and H_2_ selectivity decreased, while the CO_4_ transformation and CO discrimination rose. When the molar ratio of CO_2_/CH_4_ was 6/4, the selectivity of both carbon dioxide and water rose to 87.0% and 80.8%, respectively. This discovery provides a new perspective on the CRM reaction and advances our knowledge of how it converts when heated plasma is utilized to initiate it with no requirement for catalysis. [37].

### 2.4. Catalytic Materials for Hydrogen from Methane Decomposition

Nowadays, steam methane reforming is the primary method used to manufacture H_2_. However, this process is not ecologically friendly, releasing 10 kg of carbon dioxide for every kilogram of H_2_ produced. On the other hand, hydrogen from methane breakdown (TCD), where catalytic materials are essential, is an extremely green process that effectively creates H_2_ and valuable solid carbon with almost minimal CO_2_. Catalytic materials have been extensively studied for TCD.

Catalytic methane breakdown to produce high-purity H_2_ and high-value-added carbon nanotubes (CNTs) is a promising technology for methane resource extraction, but significant challenges are encountered in controlling CNT manufacturing. To that end, Song et al. used a structural reconfiguration strategy based on a microporous confinement process to prepare LTA zeolite-derived pebble-shell catalysts, and for the first time, molecular dynamics simulations were used to realize the tip growth of multi-walled carbon nanotubes (MWCNTs) on nickel particles, and it was confirmed that nickel particle size was closely related to the methane decomposition rate/dissolved carbon penetration/carbon nanotube growth. Furthermore, they discovered that the evolution of the nickel metal active sites paralleled the synthesis of MWCNT, demonstrating the penetration process of dissolved carbon on the nickel metal clusters, and this discovery paved the way for the construction of functionalized CDM catalysts [38]. In order to generate smaller particles from 5 to 30 nm to 5 to 10 nm, Chudakova et al. observed that adding potassium (0.25 wt% K_2_O) to the nickel catalysts altered the particle size distribution. This shift led to increased H_2_ yields. The Ni-0.25%K_2_O/Al_2_O_3_ and Ni-1%K_2_O/Al_2_O_3_ catalysts had the highest hydrogen yields of 11.6 g/gcat and 17.0 g/gcat, respectively, and the Ni-1%K_2_O/Al_2_O_3_ catalyst produced the best carbon material yield (51.0 g/gcat) [39]. In order to study the process of producing hydrogen in situ from the conversion of shale gas (methane) using microwave heating of a methane stream passing through a filled shale sample, Yan et al. carried out a number of tests. With conversion rates of 40.5% and 100% at reaction temperatures of 500 °C and 600 °C, respectively, they discovered that the methane conversion was greatly enhanced in the presence of Fe and Fe_3_O_4_ particles acting as catalysts. At lower reaction temperatures, the conversion of methane was made easier by the minerals in the shale’s catalytic action [40]. Zaghloul, Nada, and colleagues studied the catalytic pyrolysis of methane in a molten metal bubble tower to produce hydrogen gas and separable carbon. Tin was used as the base metal, and catalytically active metals like nickel and copper were added. They took into account both traditional non-catalytic reactions in the gas and parallel catalytic surface reactions on the bubble surface. The impact of temperature, pore size, and kind of molten metal on the bubble surface–volume ratio was examined and analyzed, along with the consequences of these variables on the gas-phase reactions and catalytic surface reactions within the bubbles. Finally, the best performance was obtained with the 5-weight percent Ni-Sn mixture. Xavier Jr et al. investigated the viability of CMD under the reaction conditions of graphene nanoribbon zigzag (12-ZGNR) and armchair (AGRN) edges using dispersion-corrected density functional theory (DFT). They started by looking into the desorption of H and H_2_ at 1200 K on passivated 12-ZGNR and 12-AGNR edges. On the passivated 12-ZGNR and 12-AGNR edges, the results demonstrated a very low level of H and H_2_ desorption. The most beneficial H_2_ desorption pathway is determined by the rate-determining step of hydrogen atom diffusion on the passivated edge, which has activation-free energies of 4.17 eV on 12-ZGNR and 3.45 eV on 12-AGNR. With a free-energy barrier of 1.56 eV, the 12-AGNR edge exhibits the most advantageous H_2_ desorption. This indicates that the bare-carbon active site is accessible for catalytic applications. The chemisorption of CH_4_ through direct dissociative means is not dissociative in nature. The optimal method for non-passivation of the 12-ZGNR edge is direct dissociative chemisorption, which has an activation-free energy of 0.56 eV. Furthermore, they delineated reaction pathways for the comprehensive catalytic dehydrogenation of methane on the 12-ZGNR and 12-AGNR boundaries, putting forth a mechanism via which the solid carbon that forms on the margins functions as a refreshed active site. There is a stronger tendency for regeneration at the active site on the 12-AGNR edge. Through the bare carbon edges of graphene nanoribbons, this study offers essential insights into the fabrication of carbon-based catalysts for CMD, with a methane decomposition performance comparable to that of widely used metal and bimetallic catalysts [41]. When it comes to pyrolyzing methane, molten manganese chloride (MnCl_2_) is an appealing liquid catalyst at high temperatures. Researchers Bae et al. looked at the mechanism and kinetics of the reaction for the pyrolysis of CH_4_ in mixtures of different mono- or divalent chlorides with molten MnCl_2_. A significant difference was seen between the apparent activation energy of the uncatalyzed process and the pyrolysis of CH_4_ in molten MnCl_2_ mixtures. Only the MnCl_2_-KCl mixture’s apparent activation energies were less than those of pure manganese chloride among the manganese chloride mixes. Furthermore, MnCl_2_-KCl yielded the highest crystallinity of the final solid carbon product and the greatest quantity of CH_2_*, indicating a distinct pathway for CH_4_ dehydrogenation and carbon formation in this system. This is supported by the fact that MnCl_2_-KCl forms CH_2_ at a more thermodynamically favorable rate than MnCl_2_. The pathway for the formation of graphitic carbon layers in molten MnCl_2_-KCl is more facile. Furthermore, an NVT ab initio molecular dynamics simulation shows that frequent reversible desorption and adsorption of CHx* intermediates in MnCl_2_-KCl promotes gas-phase C-C coupling. These fundamental insights into the reasons for the enhanced high-temperature decomposition of CH_4_ in MnCl_2_-KCl could be useful for the development of more reactive molten salt catalysts [42]. Copper ferrite (CoFe_2_O_4_) was used as a catalyst to break down methane, according to Alharthi, Abdulrahman I. The reaction temperature was between 800 and 900 °C, and the catalytic reaction was conducted in a fixed-bed reactor operating at atmospheric pressure and a gas flow rate of 20 to 50 mL/min. As the reaction temperature rose, the amount of methane converted and hydrogen formed increased, while catalyst stability and induction time dropped. For methane cracking, CoFe_2_O_4_’s total catalytic activity dropped at gas flow rates higher than 20 mL/min. At 20 mL/min, 900 °C, and 50 mL/min, 800 °C, the greatest carbon deposited was 70.46%.

### 2.5. Non-Oxidatively Coupled Catalytic Materials

**Figure 7:**
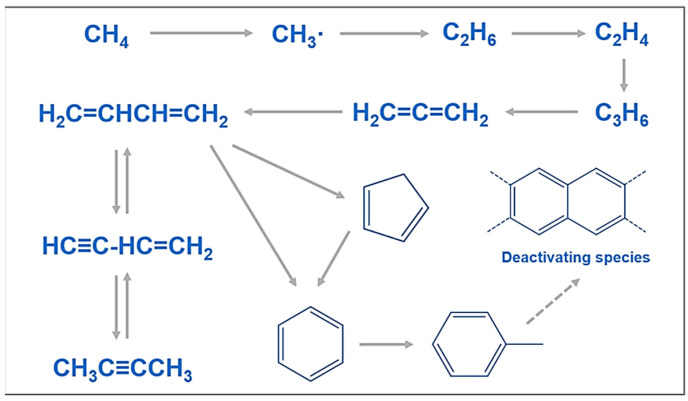
Potential pathways of reactions _f_or methane conversion using [Fe] zeolite catalysts [44].

Although the non-oxidative coupling reaction (NOCM) of methane is highly intriguing, its applications are hindered by its low catalyst stability and harsh reaction conditions (T > 800 °C), which can quickly deactivate the catalyst and cause significant coking. Metal carbides are used in a variety of catalytic processes, from thermal coupling of methane to electrochemically driven reactions, due to their platinum-like properties. Zhang et al. looked at the role carbides play in the active creation of C_2_ products during methane coupling at high temperatures. Whereas tungsten carbide (WC) loses selectivity because of the depletion of surface carbon by slow diffusion, molybdenum carbide (Mo_2_C) exhibits persistent C_2_ selectivity for an extended length of time in the gas stream due to its quick carbon diffusion kinetics. This result implies that the catalyst’s carbons are important, with the metal carbons being in charge of the production of methyl radicals, and that the nonoxidative coupling of methane is facilitated by a carbon process similar to the Mars–Van Krevelen type [43]. As seen in Figure 7, a different Zhang et al. research group created siliceous [Fe] zeolites with MFI and CHA topologies, and suggested a polyaromatic hydrocarbon reaction network. In gas-phase products, this siliceous [Fe] zeolite exhibits remarkable selectivity for ethylene and ethane (MFI > 90%, CHA > 99%). Moreover, burning coke in the air can replenish deactivated [Fe]zeolites. Oxide semiconductors coated with metals are significant photocatalysts for the nonoxidative coupling of methane [44].

Zhang et al. looked into methane nonoxidative coupling reactions with a variety of noble metal-decorated oxides (NaTaO_3_, CaTiO_3_, LiNbO_3_, and TiO_2_) adorned with Ag, Au, Pt, Pd, Cu, and Ni [45]. It is generally accepted that the active areas for CH_4_’s H extraction and C-C coupling are spatially separated; that is, metal nanoparticles complete the final C-C coupling, while NaTaO_3_ only completes the first H extraction during CH_4_ activation. Because they greatly reduce the CH_4_ dissociation energy barrier and enhance C-C interaction, precious metals predominate in the NOCM. With C_2_H_6_ yields as high as 194 mu mol g^−^^1^ h^−^^1^ in Ag/NaTaO_3_-catalyzed NOCM, silver is the metal that prefers the weak adsorption of CH_3_ intermediates at the centroid and subsequent metal-induced CC coupling among the other metals. This study provides a molecular understanding of the mechanism of CH_4_ coupling on metal-decorated photocatalysts. In the work of a different Zhang et al. team, molybdenum-doped CeO_2_ samples with isolated molybdenum sites were created using flame-jet pyrolysis. They underwent screening and evaluation for their effectiveness in the catalytic non-oxidative coupling of methane. It was discovered that the gas-phase products could have a selectivity of up to 98% for high-value-added C_2_ hydrocarbons, such as ethylene and ethane. Molybdenum-oxygen species that were separated from the produced catalysts during the procedure were reduced, changed into molybdenum (oxy)carbide species, and used as methane activation sites. This work highlights the significance of lowering the reactor’s free volume to restrict secondary gas-phase reactions, and offers insights into the development of effective catalysts for the non-oxidative coupling of methane [46]. In prior work, Ryu et al. demonstrated that Mo/HZSM-5’s methane conversion and BTX selectivity could be greatly enhanced by the addition of nickel oxide through a straightforward physical mixing process. In a more recent study, the effective diameters of promoter NiO particles (4, 22, 36, 45, and 101 nm) were assessed, and it was discovered that 36 nm was the ideal size for raising Mo/HZSM-5′s activity. Inactive NiMoO_4_ was more likely to develop when NiO particle sizes lower than 22 nm caused significant agglomeration and limited dispersion of MoCx [47]. It has been possible for Andrey A. Stepanov and associates to improve Mo/ZSM-5 catalysts for the dehydroaromatization of methane. An investigation into the catalytic impact of Mo/ZSM-5 catalysts—which depends on high-purity silica zeolites of the ZSM-5 variety with microporous and micro mesoporous structures—was carried out. It was found that the addition of carbon black during the ZSM-5 zeolite synthesis stage did not cause structural changes, and the generated samples exhibited 100% crystallinity. Throughout the methane dehydrogenation process, the Mo/ZSM-5 catalysts’ stability and activity are increased by their capacity to take on a microporous form. The 4.0 percent Mo/ZSM-5 catalyst developed utilizing zeolite synthesized from 1.0% carbon black displayed the greatest transformation of methane, achieving 13 percent after 20 min, and benzene synthesis, reaching 7.0% [48]. In order to create 4%Mo/ZSM-5 catalysts for the non-oxidative conversion of methane to aromatic hydrocarbons, Stepanov et al. examined the impact of secondary mesoporous structure formation in ZSM-5 zeolites on the catalytic characteristics of those catalysts. Carbon black was added to zeolites that were manufactured, zeolites treated with aqueous citric acid, and zeolites featuring a microporous structure without binders, in order to create 4%Mo/ZSM-5 catalysts. It was demonstrated that molybdenum modification of zeolites with microporous structure reduced the concentration and strength of the strong acid centers in the aromatization of methane, independent of the synthesis method; the most efficient treatment of the 4%Mo/ZSM-5 catalyst was with a 0.3 N citric acid solution [49].

## 3. Toluene Handling Materials

### 3.1. Toluene Adsorption Materials 

**Figure 8:**
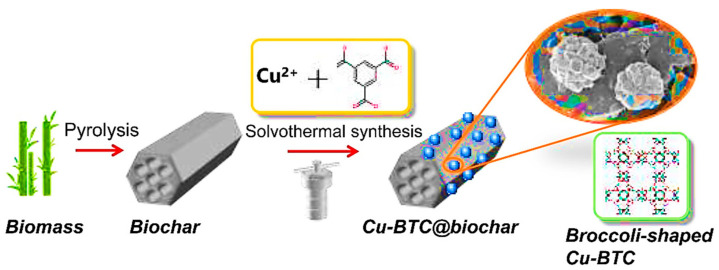
Diagram showing the steps involved in creating Cu-BTC@biochar [50].

Eliminating toluene is crucial for lowering air pollution, since it is a common example of an aromatic volatile organic compound (VOC). Adsorption of volatile organic compounds (VOCs) from gases, including toluene, is a proven method that is frequently employed in industrial production processes to eliminate VOCs while reusing them for future use. As shown in Figure 8, Zhang et al. synthesized a size-controlled, mallow-like copper-carbon tetrachloride-loaded biochar composite (Cu-BTC@biochar) using a simple one-pot method. The adsorption rate of Cu-BTC@biochar on toluene, a typical component of volatile organic compounds (VOCs), could reach 501.8 mg/g and 88.8 mg/g at medium and high temperatures (60 and 150 °C), respectively [50].

**Figure 9:**
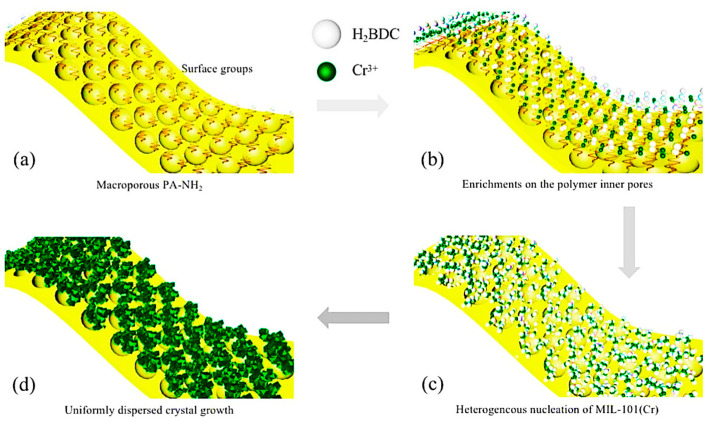
Diagram showing the limited development method of MIL-101(Cr) nanocrystals on the PA-NH_2_ substrate’s macroporous surface [52].

The pore distribution properties of porous adsorbent materials are among their most significant features, and many studies have looked at how to accurately and conveniently tailor usable porous carbons for the adsorption of volatile organic compounds (VOCs). Because of this, Wang et al. produced porous carbon for model experiments using precursors with various lignocellulose mass ratios. Additionally, they verified the pore shape and distribution properties of the porous carbon by using bacterial targeting of bagasse decomposition to validate the application of these mechanisms in real-world biomass materials. While the microvolume of the mesopores exhibited the opposite tendency, the microvolume of the ultramicro pores decreased as the cellulose concentration decreased. Both BACs-36 and BACs-48 showed excellent toluene adsorption capabilities at low concentrations of 635 mg/g and across ten cycles, respectively. Poor humidity stability in the presence of air humidity and the need for powder molding have greatly impeded the removal of volatile organic compounds (VOCs) from the metal-organic framework (MOF) MIL-101(Cr) powders [51]. Zhang et al. employed an in situ growth confinement technique to create the hydrophobic composite P-MIL-101(Cr)@PA-NH_2_ in order to address this problem. On the macroporous surfaces of PA-NH_2_ substrates, the MIL-101(Cr) nanocrystals’ confinement growth method is schematically shown in Figure 9. The material’s working capacity was 6.3 times larger than that of the original MIL-101(Cr) powder at 1000 mg/m^3^ toluene and 50% relative humidity. Some 81.1% of the material’s initial adsorption capacity remained after 30 days in a humid environment. The molecular sieve has problems with poor moisture resistance, high-temperature desorption, and a low-temperature desorption rate when it comes to purifying volatile organic compounds [52].

Zhou et al., on the other hand, developed a microporous bifunctional system for adsorption/catalysis by changing the mass proportion of Pt/microporous silica (Pt/MS) from 1.0 wt% to 3.5 wt% in order to achieve the best adsorption/catalytic performance for trace toluene. They additionally hydrophobically modified the created Pt/MS catalyst to make Pt/MS-H in order to boost the Pt/MS catalyst’s resistance to water vapor. At a desorption temperature of 60 °C, 2.0 Pt/MS-H exhibited an optimal saturation adsorption capacity of 56.09 mg/g for toluene. Following five cycles of adsorption and desorption, the toluene adsorption capacity was kept at roughly 95%, with a good peak smoothing effect. Trace toluene conversion capacity at T90 = 149 and DEG; C was optimal. For volatile organic molecules, it can be a powerful auxiliary material when used in conjunction with adsorption–catalysis [53]. The removal of toluene from laboratory to industrial scale utilizing a unique thermal mass exchanger was evaluated for the first time by Villarim et al., who also clarified the molecular process of absorption. They ascertained the absorption capacities and vapor–liquid partition coefficients (K) of three benzhydrols, namely propylene glycol, acetic acid, and their aqueous mixes. They discovered that in the solvents under study, the absorption of volatile organic molecules reduced as the concentration of water rose. Overall though, the water/benzyl alcohol mixes (60:40 wt%) show a high absorption capacity that is comparable to other organic solvents, and has considerable promise for the treatment of industrial air contaminated with toluene [54].

### 3.2. Catalytic Oxidizing Materials

Catalytic oxidation has been extensively studied as an efficient, resource-aware method of removing toluene from indoor air and industrial exhaust gases without additional treatment. Transition metal-functionalized γ-alumina carriers are widely used as industrial catalysts for the complete oxidation of volatile organic molecules at elevated temperatures, and Zumbar et al. achieved full oxidation activity of toluene at lower temperatures (200–380 °C) by rationally designing bimetallic CuFe-γ-alumina catalysts [55].

Fe–oxalate complexes under UV and visible light irradiation are the basis of a novel technique Zhao et al. devised for the effective recovery of platinum. Under UV radiation, the process can recover 98.9% of platinum in 30 min, and in sunshine, it can recover 95.6% of platinum in 8 h. This work reduces the cost of producing new catalysts by developing an environmentally responsible method of recycling platinum from discarded catalysts [56]. Conversely, Zhou et al. synthesized a variety of ceria-based high-entropy oxide catalysts using a solid-state reaction method. Among these, the Ce-HEO-T sample had a 100% conversion rate when it came to catalyzing the oxidation of toluene at 328 °C. After the addition of varying amounts of gold, Ce-HEO-500, which has the lowest toluene oxidation temperature, can perform even better. When compared to the Ce-HEO-500 support, the somewhat gold-containing Au/Ce-HEO-500 sample demonstrated about 70 °C lower toluene combustion, at 260 °C. It also showed good stability, converting 98% of the toluene over 60 h. Furthermore, demonstrating exceptional water resistance, the toluene conversion at 5% H_2_O vapor stayed constant, and even significantly enhanced during the conversion in dry air [57].

To maximize lattice oxygen activation of pristine CoMnOx (CMO-E0) during toluene oxidation by acid treatment, Wang et al. synthesized nanostructured cobalt–manganese oxides (CoMnOx) with cationic defects (CMO-Ex, where x represents the acid concentration). The maximum toluene catalytic degradation activity was shown by the CMO-E_0.05_ sample that had been adjusted with an ideal manganese and cobalt defect content. Furthermore, the CMO-E_0.05_ sample exhibits superior water resistance and catalytic stability [58]. Alpha-MnO_2_ doped with four metals (Cu, Ce, Co, and Fe) was synthesized by Jiang et al. using redox co-precipitation. MnCu exhibited the maximum activity, with 90% toluene conversion at a temperature of 224 °C and a weight-hourly space velocity of 30,000 mL·g^−1^·h^−1^. The most active was MnFe, which had a lot of surface defects and a relatively active surface lattice of oxygen, which sped up toluene adsorption and activation. The inadequate migration ability of lattice oxygen prevented the deep oxidation of toluene. Furthermore, the catalyst surface may be covered with adsorbed toluene and certain intermediates, which would prevent toluene from oxidizing continuously. Consequently, when designing mixed-oxide catalysts based on manganese dioxide, the profound oxidation of toluene is far more crucial than adsorption [59]. As adsorbents and catalysts, Zhou et al. synthesized a range of Mn/ZSM-5 catalysts with varying Mn concentrations (2, 4, and 6 wt%) to examine the regeneration performance of toluene adsorption and ozone-catalyzed oxidation at ambient temperature. Toluene was oxidized using a catalyst containing 4 weight percent Mn/ZSM-5, with a 90% conversion rate at 30 °C. Good toluene adsorption capability was still maintained by the 4 weight percent Mn/ZSM-5 after four successive adsorption–ozone cycles [60]. CexMn1-x O_2_ catalysts were synthesized by Zhou et al. using the sol-gel method, and their catalytic activity for the oxidation of toluene was shown to be better than those of single oxides. The catalysts were prepared with different mixing ratios. The toluene oxidation reaction’s ring-opening reaction was aided by the doped manganese’s increased oxygen vacancy count and capacity to activate aromatic rings. Due to its readily available nature and low energy usage, photocatalytic technology has garnered significant attention for the removal of toluene. However, heat is produced by light, and when temperature rises, pollutants may desorb from the catalyst, making deterioration less likely [61]. Accordingly, Yu et al. conducted a detailed analysis of toluene adsorption on UiO-66 (Zr) and postulated a synergistic interaction between heat-induced adsorption and photocatalysis. According to the findings, UiO-66 (Zr) had a greater ability to adsorb toluene at higher temperatures. At the ideal temperature of 30 °C, the removal rate of toluene was 69.6% [62].

### 3.3. Catalytic Materials for Chemical Cycle Reforming

**Figure 10:**
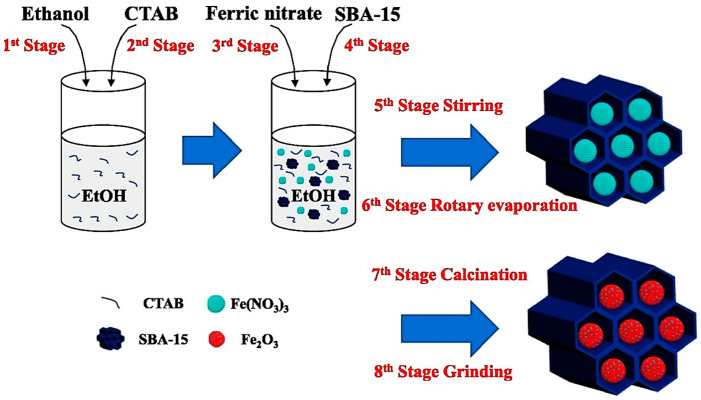
Fe_2_O_3_@SBA-15 schematic diagram created by wet impregnation [64].

Syngas with a high hydrogen content is a valuable industrial feedstock and a sustainable energy source. The chemical equilibrium of the water–gas shift reaction limits the composition of the syngas produced in the traditional chemical looping steam reforming (CLSR) process. A two-step chemical cycle-reforming (TS-CLR) process can reduce the equilibrium limitation and generate syngas with larger CO and H_2_ concentrations, according to research by Xu et al. The important components in this are oxygen carriers with outstanding cycle performance and good partial oxidation performance [63]. In order to enhance the cycling and partial oxidation ability of the oxygen carrier, Mao et al. proposed an embedding technique to modify the microreactor environment of the oxygen source carrier. As shown in Figure 10, they made Fe_2_O_3_@SBA-15 catalysts by embedding Fe_2_O_3_ into SBA-15 via wet impregnation, and investigated its catalytic role in the toluene chemical cycle-reforming reaction [64].

**Figure 11:**
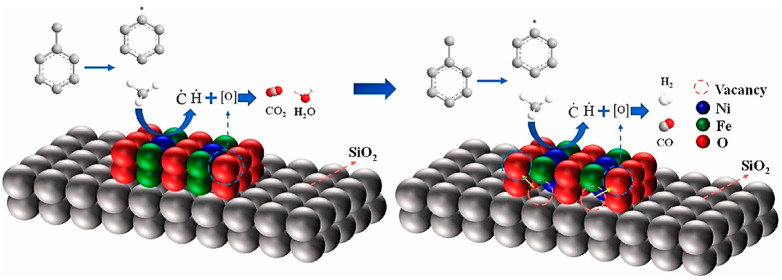
Chemical cycle reforming of NiFe_2_O_4_@SBA-15 with toluene: a reaction route diagram [66].

It was found that catalytic cracking and partial oxidation are the two steps that make up the toluene chemical cycle reforming process, where the first stage mainly produces syngas and the second stage produces coke and H_2_. Comparing Fe_2_O_3_@SBA-15 to pure Fe_2_O_3_, the CO selectivity rose from 25.9% to 96.2%. Significant improvements were made in the lattice oxygen usage and toluene conversion. The toluene conversion dropped by just 1.9% after ten cycles. By embedding metal oxides in molecular sieves, Liu et al. developed NiO@SBA-15, Fe_2_O_3_@SBA-15, and NiFe_2_O_4_@SBA-15 based on the decoupling method of the biomass chemical cycle gasification process. They investigated catalytic toluene chemical cycle regeneration. The results showed that NiFe_2_O_4_@SBA-15-catalyzed toluene could reach a highest conversion rate of 93.4% with a CO selectivity of 80.7%. By enhancing the dispersion and nanocrystallization of metal oxides in the oxygen carrier, the embedding technique can substantially decrease sintering. The ideal weight–time rate was 1.168 h^−^^1^, and the ideal reaction temperature was 750 °C. Over 10 testing cycles, the average toluene conversion was 95.34%, and the moderate CO selectivity was 94.83% [65]. Zhang et al. also created NiFe_2_O_4_@SBA-15 by employing an impregnation-based embedding technique. At 750 °C, methane and toluene underwent a chemical cycle-reforming process. Figure 11 depicts the reaction’s route diagram. Toluene conversion was 97.5%. The methane and toluene chemical cycle reaction was conducted using the impregnation method [66].

**Figure 12:**
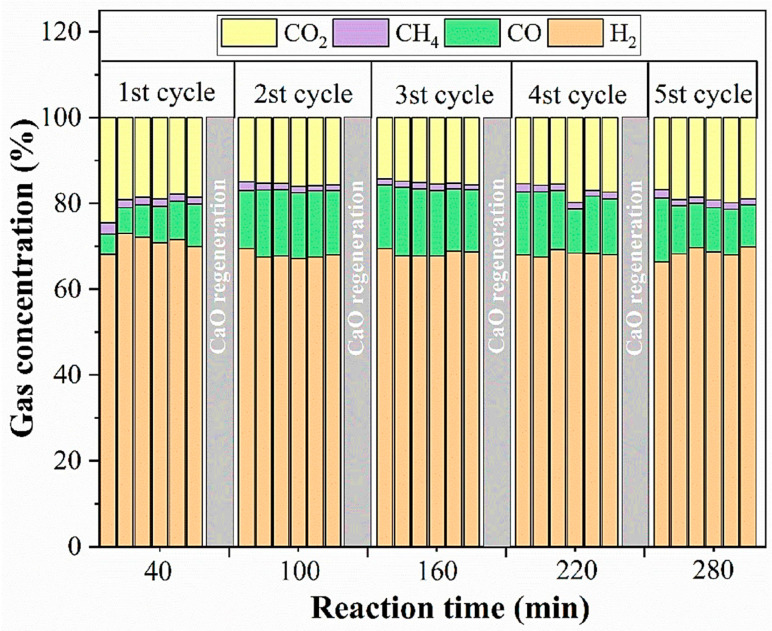
The concentration of syngas during multicycle toluene conversion with a Ca-Al-Fe absorber present (single column image) [69].

Sun and colleagues introduced a novel approach for biomass pyrolysis of volatiles: chemical loop reforming (CLR). The method is based on a decoupling technique, and its reaction kinetics and cycle performance were investigated for the toluene CLR process on LaFe_0.6_Co_0.4_O_3_@SBA-15 OC. They discovered that the toluene conversion rose from 52.3% to 79.7%, the CO selectivity climbed from 57.0% to 87.4%, and the oxygen release (OR) increased by 100% when LaFeO_3_ was encapsulated in SBA-15. Improved reaction performance, decreased sintering, and enhanced dispersion of the metal oxides were the outcomes of the encapsulation effect. Co-inclusion produced the best results, with a toluene conversion of 81.6% and a CO selectivity of 96.8%. During ten testing cycles, LaFe_0.6_Co_0.4_O_3_@SBA-15’s toluene conversion and CO selectivity stayed between 90.0% and 92.0% and 93.0% and 96.0%, respectively. This work offers recommendations for the use of organic compounds (OC) for the biomass pyrolysis volatile chemical cycle-reforming process [67]. Luo et al. used 6Ni, 4Ni_2_Cu, and 4Ni_2_Fe as oxygen carriers and toluene as a tar model molecule. They used different temperatures and water vapor–carbon molar ratios (S/C) to study the chemical cycle reforming reaction of toluene. It was found that the metal oxygen carriers acted as both oxidizing and catalyzing agents in the chemical cycle reforming of toluene. After ten cycles, 4Ni_2_Fe outperformed the other two oxygen carriers in terms of carbon deposition resistance, stability, and regeneration [68]. Rong et al. developed a two-step sol-gel process to manufacture Ca-Al-Fe, a hybrid adsorbent/catalyst for calcium cycle gasification (CLG) based on calcium oxide. With the least amount of coke deposited, the highest average H_2_ production and an average hydrogen concentration of roughly 68.8% throughout five toluene-reforming cycles were demonstrated by Ca-Al-Fe. The percentage of hydrogen was roughly 68.8% on average. This is roughly 26.41% greater than the typical CaO conversion. Figure 12 displays the syngas concentration throughout the multi-cycle toluene conversion [69].

### 3.4. Degradation of Catalytic Materials

**Figure 13:**
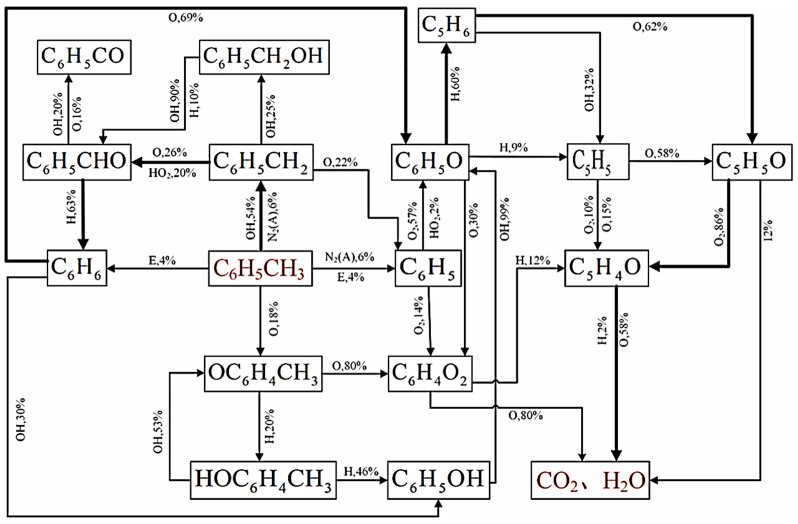
Degradation route of toluene and the ratio of products produced at each stage of the route [70].

VOCs can be broken down using a very promising green technique called non-thermal plasma (NTP). In accordance with the increasing amount of research being carried out in this field, Yue et al. explored the mechanism of toluene degradation in air/H_2_O dielectric barrier discharge (DBD) plasma, finding that at P = 115 W, Cin, toluene = 1000 ppm, the degradation efficiency of toluene was >82%. As illustrated in Figure 13, the breakdown mechanism of toluene is as follows: toluene → phenyl → benzaldehyde → benzene → phenoxy → cyclopentadiene → polycarbonate/alkyne → CO_2_/H_2_O. This advances the development of non-thermal plasma breakdown of volatile organic molecules and offers fresh insights into the plasma-catalyzed process [70].

One important use of photocatalysis is the breakdown of volatile organic molecules. Liu et al. used a straightforward sol-gel technique to adsorb vanadium–nitrogen co-doped TiO_2_ onto a honeycomb ceramic (V/N-TiO_2_@HC), creating a continuous-flow photocatalytic degradation reactor. The insertion of V/N dopants decreased the band gap and expanded the light absorption range of TiO_2_, resulting in a continuous and nearly full photocatalytic degradation of toluene in this reactor. The reactor’s abundant ordered pores in HC facilitated the mass transport of toluene. The photocatalyst’s reusable application was made possible by HC’s distinct and sturdy structure. The toluene gas degradation rate reached 97.8% and stayed at 96.7% over 24 cycles of photocatalytic degradation [71]. Using atmospheric surface photovoltaic (ASPV) spectroscopy, After heating TiO_2_ in a hydrogen environment to produce more bridging hydroxyl groups (OHBs), Zhong et al. investigated the effects of surface site modifications on the transfer of charge carriers generated by photosynthesis to the reacting substances (O_2_, H_2_O, and toluene). The researchers found that treatment with hydrogen produced gap transmission channels for toluene and water and electron transmission routes for oxygen, and reduced H_2_O interference with oxygen’s ability to use electrons. The hydrogen found in the freshly formed OHB changed the distribution of electrons on the surface of TiO_2_ by introducing electrons. The reaction was accelerated by the OHB and the surrounding Ti4-x ions, which changed the adsorbed form of H_2_O and helped transport electrons to oxygen (O_2_) and gaps to toluene. Due to the great photothermal conversion ability and good thermocatalytic activity of transition metal oxides, it is possible to further enhance their photothermal catalytic capacity by logically triggering the photoelectric effect of semiconductors [72]. On this basis, Zhao et al. synthesized Mn_3_O_4_/Co_3_O_4_ composites with S-type heterojunctions for the photothermal catalytic degradation of toluene under ultraviolet–visible (UV-Vis) light. Under UV-Vis irradiation, the rapid electron transfer between the surfaces of the Mn_3_O_4_/Co_3_O_4_ composites promoted the generation of more reactive radicals, and the energy band bending and the intrinsic electric field at the Mn_3_O_4_/Co_3_O_4_ interface enhanced the photogenerated carrier transport pathway and maintained high redox potential. The elimination of toluene by Mn_3_O_4_/Co_3_O_4_ was improved from 53.3% and 47.5% to 74.7% compared to single metal oxides, respectively.

This work provides important guidance for the creation of effective narrow-band semiconductor heterojunction photothermal catalysts [73].

Toluene degradation by heterojunctions composed of rutile–rutile TiO_2_ is thought to be a successful method; however, photogenerated electron usage is still inadequate. In order to enhance the photocatalyst’s catalytic efficiency, Zhang et al. created a type II heterojunction using rutile-coated Lavoisier Institute material (MIL-101). It was discovered that in addition to the transfer of photogenerated electrons to anatase’s oxygen vacancies, which encourages the production of oxygen-containing radicals, the enhancement of the photocatalytic performance also depends on the anatase’s ability to encapsulate MIL-101, its capacity to absorb light, and the contact area between the two heterojunctions. The substance illustrates the complementary nature of heterojunction and heterojunction design, offering a theoretical foundation for their use in the breakdown of volatile organic molecules [74].

**Figure 14:**
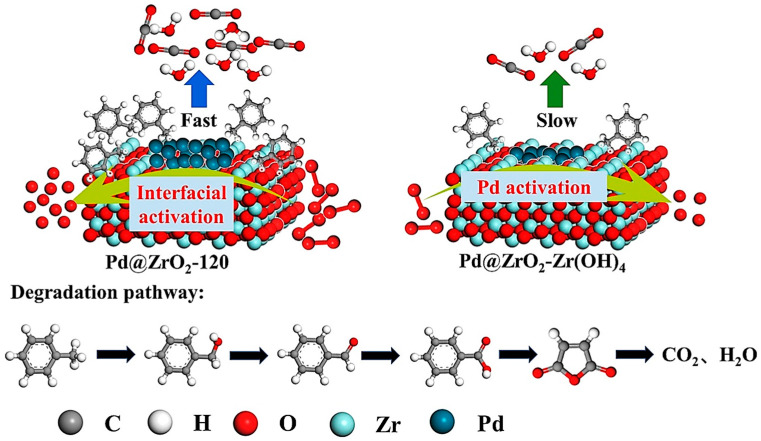
Mechanism of degradation of Pd@ZrO_2_-120 and Pd@ZrO_2_-Zr(OH)_4_-oxidized toluene [75].

One efficient method to encourage the deep decomposition of volatile organic compounds (VOCs) is to modify the interactions between the metal and the support. In order to encourage the deep degradation of toluene, Bi et al. produced a Pd@ZrO_2_ catalyst utilizing the Zr-based metal-organic framework (MOF) Pd@UiO-66, grown in situ, as a precursor. Figure 14 illustrates the degradation pathway [75].

When Pd@ZrO_2_-Zr(OH)_4_ was synthesized using Zr(OH)_4_ as a precursor, MOF-derived Pd@ZrO_2_ catalysts with varying calcination periods performed better in terms of toluene degradation, water resistance, and stability. Toluene’s profound breakdown into CO_2_ and H_2_O happened more quickly. This work offers recommendations for optimizing interfacial interactions to enhance the deep degradation of volatile organic compounds (VOCs) using MOF-derived catalysts. Zhu and colleagues built a coaxial dielectric barrier discharge (DBD) reactor and loaded a sequence of Cu-MnO_2_/gamma-Al_2_O_3_ catalysts into the plasma device via impregnation and redox processes to break down a toluene and o-xylene combination. The results showed that the Cu-doped MnO_2_ catalysts drastically reduced the production of byproducts while also considerably improving CO_2_ selectivity and pollutant removal. Toluene and o-xylene were both able to achieve a 100% removal rate and 92.73% CO_2_ selectivity, with Cu_0.15_Mn/gamma-Al_2_O_3_ exhibiting the greatest removal rate among them. This work offers theoretical direction and a useful foundation for the use of mixed benzene series volatile organic compounds (VOCs) catalyzed by DBD [76].

One efficient method to encourage the catalytic breakdown of volatile organic compounds (VOCs) is to modify the strength of the metal–oxygen connection to activate surface lattice oxygen (O-latt). Following the Mars–van Krevelen mechanism, Zhu et al. demonstrated that toluene could be oxidized by fast dehydrogenation of methyl groups with the help of highly active surface O-latt, followed by additional ring opening and deep mineralization to CO_2_. This work offers a fresh approach to investigating interfacially enhanced transition metal catalysts for effective VOC abatement and surface O-latt activation [77].

**Figure 15:**
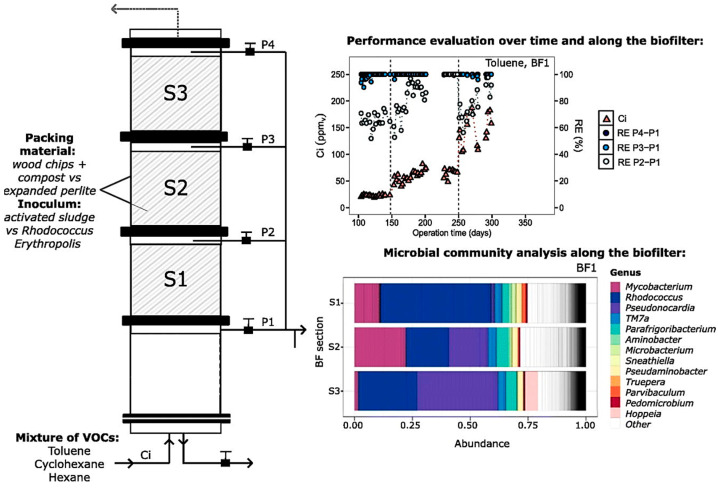
The experiment’s flow chart, performance assessments along the biofilter and over time, and a microbiological community study along the biofilter [78].

Biofiltration is an efficient and economical treatment technology for the removal of volatile organic compounds (VOCs) from exhaust gas streams. Pineda et al. evaluated the effect of inoculum type on the removal of toluene, cyclohexane, and n-hexane mixtures in three biofilters (BF_1_, BF_2_, BF_3_), and the reaction flow chart and related data are shown in Figure 15 [78].

The three biofilter inoculum types were BF_1_: a compost and wood chip mixture and *Erythrobacter* spp.; BF_2_: a compost and wood chip mixture and acclimatized activated sludge with *Erythrobacter* spp.; and BF_3_: an expanded perlite and *Erythrobacter* spp. The three biofilters were operated for 374 days at different inlet loads and empty bed residence times. It was found that toluene was removed first, followed by cyclohexane, and hexane was removed last. At each stage of operation, BF_2_ outperformed BF_1_ and BF_3_, with average maximum removal capacities as follows: toluene: 21 ± 3 g·m^−^^3^·h^−^^1^; cyclohexane: 11 ± 2 g·m^−^^3^·h^−^^1^; and hexane: 6.2 ± 0.9 g·m^−^^3^·h^−^^1^. Despite the differences in inoculum, the dominant microorganisms in all the biofilters were Rhodococcus, Mycobacterium, and Hexane. Rhodococcus, Mycobacterium, and Pseudonocardia genera; only the relative abundance was different. This study provides new ideas for the removal of VOCs.

From saline soil in Wadi An Natrun, Barghoth et al. isolated sixteen pure halophilic bacterial isolates capable of producing energy and carbon from toluene as the only source. Isolate M_7_ had the best development and most similar characteristics out of all of these isolates. With 99% similarity to Exiguobacterium mexicanum, strain M7 belongs to the Exiguobacterium genus. With toluene serving as the only carbon source, strain M_7_ exhibited good growth characteristics over a broad temperature range of 20–40 °C, pH values of 5–9, and salinity concentrations of 2.5–10%, *w*/*v*. The ideal growth conditions were found to be 35 °C and pH values of 8–9% and 5%, respectively. In 48 h, strain M_7_ broke down 88.32% of toluene under ideal circumstances. The present results suggest that strain M_7_ can be used as a biotechnological tool for the treatment of toluene waste [79].

## 4. Summary and Outlook

A variety of methods and materials are available here for the treatment of methane and toluene. Adsorption, catalytic oxidation, reforming, degradation, and other technologies all allow for the removal and resource utilization of methane and toluene. Many of these materials can also be very environmentally friendly and safe for the treatment and resource use of methane and toluene, and many can achieve conversion rates of 90% or more and remain active after reuse more than 10 times. However, there are still some areas for improvement. Firstly, certain auxiliary materials for the treatment of methane and toluene often need to function at high temperatures, high pressures, precious metals, etc., which undoubtedly requires the use of more energy and resources. Therefore, we need to further optimize these conditions and develop more inexpensive catalytic materials to reduce the use of energy and expensive resources.

Second, although there has been more study on materials for treating single gases, we must carry out more research into materials for treating mixed gases that cause pollution.

Thirdly, a lot of materials are only appropriate for use in laboratories, and cannot be used in industrial production or application. This is significant because if industrial production and application can be achieved, people will likely be able to deal with these two gases on a large scale, which will likely have a positive effect on both the environment and human health.

All things considered, future studies on related materials can begin with the aforementioned three principles and be refined regularly to provide more direction and support for the preservation of the environment worldwide, as well as the economical use of energy.

## Data Availability

No new data were created in this study.
